# Supplementary figures and images for: Telemonitoring at scale for hypertension in primary care: An implementation study
Source: PLoS Med. 2020 Jun 17;17(6):e1003124. doi: 10.1371/journal.pmed.1003124 (PMC7299318; doi:10.1371/journal.pmed.1003124)

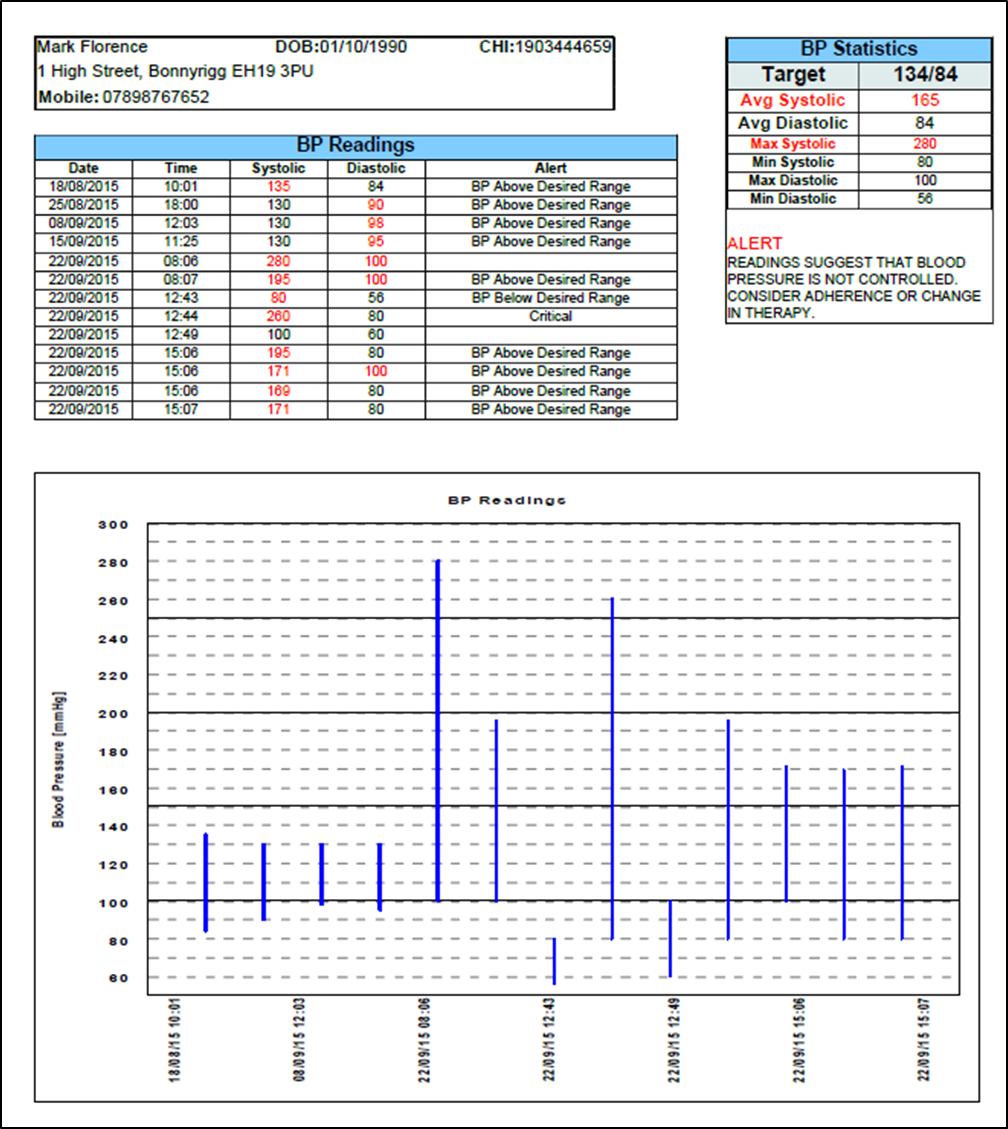

Supplement: S2 Fig — (TIF) [file pmed.1003124.s002.tif]

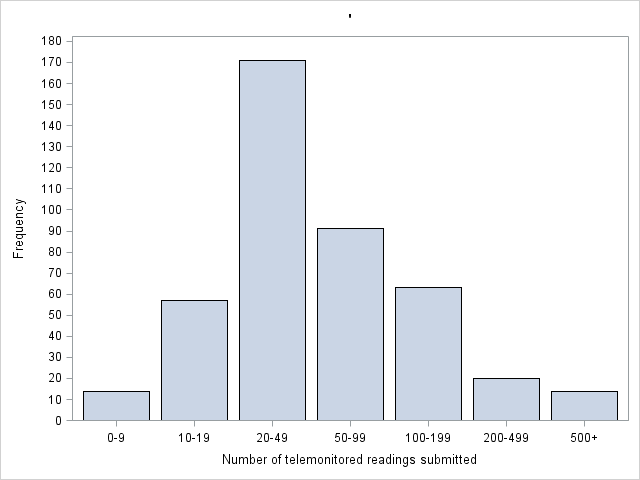

Supplement: S4 Fig — (TIF) [file pmed.1003124.s004.tif]

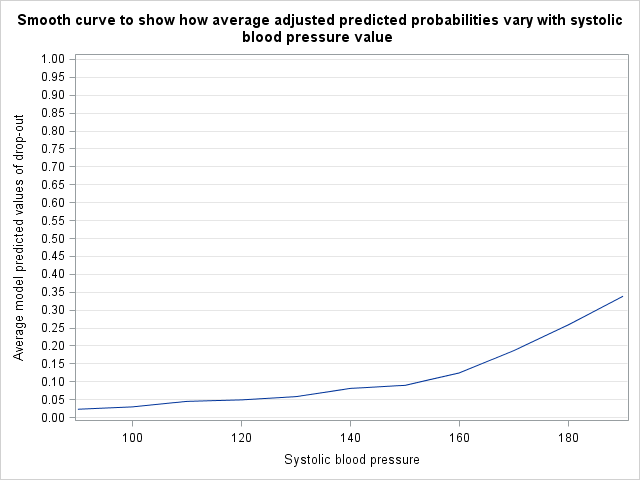

Supplement: S5 Fig — (TIF) [file pmed.1003124.s005.tif]

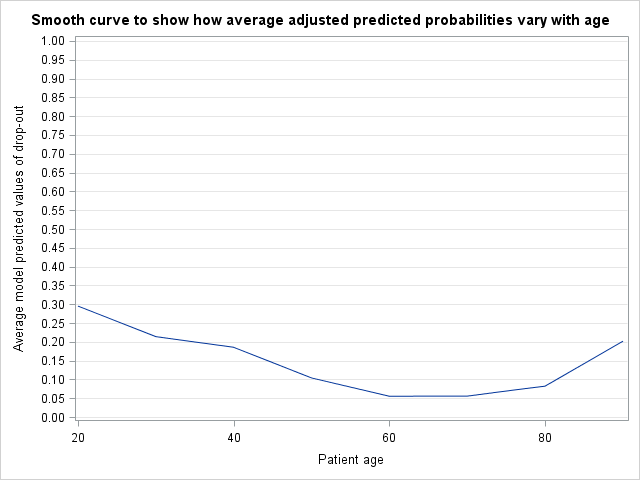

Supplement: S6 Fig — (TIF) [file pmed.1003124.s006.tif]

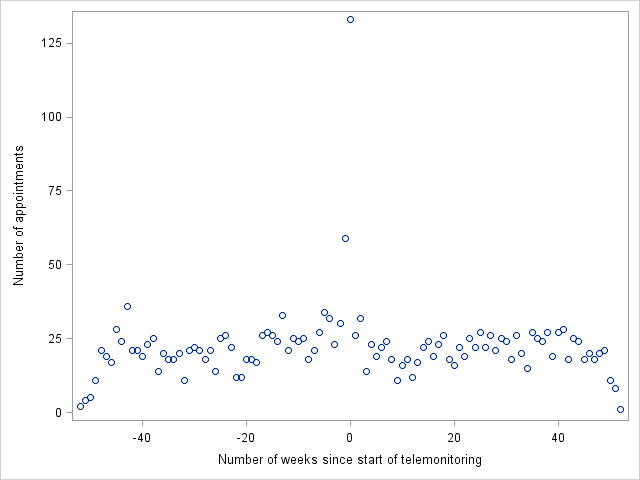

Supplement: S7 Fig — (TIF) [file pmed.1003124.s007.tif]
