# Supplementary material for: Telemonitoring at scale for hypertension in primary care: An implementation study
Source: PLoS Med. 2020 Jun 17;17(6):e1003124. doi: 10.1371/journal.pmed.1003124 (PMC7299318; doi:10.1371/journal.pmed.1003124)
Supplement: S1 Table — (DOCX) [file pmed.1003124.s010.docx]

**S1 Table. Barriers identified and proposed solutions to implementing telemonitoring at scale**

| **Barrier/facilitator identified from previous work** | **How the implementation strategy addressed barriers and optimised facilitators** |
| --- | --- |
| Intervention needs to integrate with existing systems and routines | Development of a Scale-Up BP system for feeding back the patients’ summarised data through the practices’ usual data management system (Docman) so reviewing incoming data required no additional activity. |
| Clinicians need to be convinced of the scale of the problem, and the strength of the evidence that the telemonitoring intervention could both be clinically effective and ideally reducing their workload. | Talks at professional meetings, circulars to clinicians, meetings with the practice teams provided a consistent message, highlighting the trial evidence that telemonitoring improved BP control, and emphasising the potential time saving nature of the telemonitoring intervention and the easy integration with their existing routines. |
| Practice teams need training and support as they learn to adopt the new systems | Trainers went to practices to train nurses and health care assistants who were also provided with written information. A helpline and re-training was easily available if required (e.g. if there was a change of staff) |
| Equipment needed to be provided for patients to overcome concerns about inequity | Practices were provided with sphygmomanometers to distribute to patients, along with explanatory leaflets for patients. The feedback system was text-based so worked on the most basic of mobile phones. |
| The use of local champions [1] | We recruited ‘tech friendly’ primary care leads to be among the first to adopt the technology. We expected them to understand the developmental nature of the technology, be tolerant of early ‘bugs’ and to contribute to the development of the implementation strategy and the iterative process of scaling up of the intervention. |
| Practices wanted to adapt how they implemented the intervention in their practice – and to ‘try and adapt’ over time | We interviewed early adopters/champions at two time points following implementation and disseminated the learnings to later adopters. Continuous feedback from practices was encouraged with suggestions for improvements incorporated in the implementation strategy. |
| Practices wanted to use different methods to enrol patients and chose which patients to target (controlled/uncontrolled; complicated/uncomplicated). | We suggested and provided practical support for some strategies (waiting room promotional materials, opportunistic recruitment on routine attendance, systematic mailing of patients on the hypertension register, group evening events). Experience of successful implementers was fed back to other practices via the training team, newsletters, word of mouth. |

[1] Miech EJ, Rattray NA, Flanagan ME, Damschroder L, Schmid AA, Damush TM. Inside help: An integrative review of champions in healthcare-related implementation. SAGE Open Med. 2018;6:2050312118773261. doi: 10.1177/2050312118773261. PubMed PMID: 29796266; PubMed Central PMCID: PMCPMC5960847.
