# Supplementary material for: Telemonitoring at scale for hypertension in primary care: An implementation study
Source: PLoS Med. 2020 Jun 17;17(6):e1003124. doi: 10.1371/journal.pmed.1003124 (PMC7299318; doi:10.1371/journal.pmed.1003124)
Supplement: S2 Table — (DOCX) [file pmed.1003124.s011.docx]

**S2 Table: Logistic regression results for the analysis of attrition**

| **Effect** | **Odds Ratio** | **95% Confidence Limits** | | **P-value** |
| --- | --- | --- | --- | --- |
| **Female** | 0.832 | 0.440 | 1.574 | 0.572 |
| **Systolic blood pressure** | 1.029 | 1.009 | 1.049 | 0.005 |
| **SIMD 5+** | 0.973 | 0.945 | 1.003 | 0.559 |
| **Age** | 0.792 | 0.363 | 1.732 | 0.074 |
