# Supplementary material for: Telemonitoring at scale for hypertension in primary care: An implementation study
Source: PLoS Med. 2020 Jun 17;17(6):e1003124. doi: 10.1371/journal.pmed.1003124 (PMC7299318; doi:10.1371/journal.pmed.1003124)
Supplement: S3 Table — Excludes 2 weeks either side of the anchor point. (DOCX) [file pmed.1003124.s012.docx]

**S3 Table. Number of all appointed activities and face-to-face appointments in telemonitored group y and comparator group, by age, sex, deprivation status and starting BP. Excluding two weeks either side of the start point.**

|  | **Telemonitoring n=118** | | | **Comparator group n=9061** | | |
| --- | --- | --- | --- | --- | --- | --- |
| **Number of all appointments** | **One year before** | **One year after** | **% Change** | **One year before** | **One year**  **after** | **% Change** |
| Total | 1100 | 1068 | -2.9 | 97916 | 94017 | -4.0 |
| Age<65 | 589 | 527 | -10.5 | 24566 | 23902 | -2.7 |
| Age 65+ | 511 | 541 | 5.9 | 67178 | 65351 | -2.7 |
| Male | 656 | 699 | 6.6 | 41412 | 39711 | -4.1 |
| Female | 444 | 369 | -16.9 | 56504 | 54306 | -3.9 |
| SIMD<5 | 185 | 119 | -35.7 | 23242 | 21601 | -7.1 |
| SIMD 5+ | 869 | 926 | 6.6 | 73742 | 71390 | -3.2 |
| SBP<135 | 432 | 369 | -14.6 | 33406 | 34935 | -4.6 |
| SBP 135 or above | 659 | 686 | 4.1 | 48726 | 51893 | 6.5 |

| **Number of face-to-face appointments)** | **One year before** | **One year after** | **% Change** | **One year before** | **One year**  **after** | **% Change** |
| --- | --- | --- | --- | --- | --- | --- |
| Total | 763 | 619 | -18.9 | 52138 | 46617 | -10.6 |
| Age<65 | 405 | 308 | -24.0 | 15388 | 14162 | -8.0 |
| Age 65+ | 358 | 311 | -13.1 | 35655 | 31748 | -11.0 |
| Male | 458 | 394 | -14.0 | 23755 | 21261 | -10.5 |
| Female | 305 | 225 | -26.2 | 28383 | 25356 | -10.7 |
| SIMD<5 | 118 | 73 | -38.1 | 11388 | 10235 | -10.1 |
| SIMD 5+ | 608 | 532 | -12.5 | 40249 | 35882 | -10.8 |
| SBP<135 | 301 | 226 | -24.9 | 18175 | 16772 | -7.7 |
| SBP 135 or above | 456 | 384 | -15.8 | 28539 | 27493 | -3.7 |
