# Supplementary material for: Telemonitoring at scale for hypertension in primary care: An implementation study
Source: PLoS Med. 2020 Jun 17;17(6):e1003124. doi: 10.1371/journal.pmed.1003124 (PMC7299318; doi:10.1371/journal.pmed.1003124)
Supplement: S4 Table — (DOCX) [file pmed.1003124.s013.docx]

**S4 Table : Adjusted mean differences of resource use outcomes for telemonitoring versus control from linear mixed effects models, adjusting for site (as a random effect), gender, initial systolic blood pressure (SBP145, SBP 135-145, SBP<135), Age (<65 versus 65+) (N=7429)**

|  | **Adjusted mean difference (Telemonitoring – Control)** | **95% confidence interval** | **P-value** |
| --- | --- | --- | --- |
| **Reduction in total number of consultations** | 1.288 | -0.436 to 3.013 | 0.143 |
| **Reduction in total number of surgery consultations** | 0.839 | -0.149 to 1.827 | 0.096 |
| **Reduction in total consultation time (minutes)** | 16.126 | 0.135 to 32.117 | 0.048 |
| **Reduction in total surgery consultation time (minutes)** | 12.725 | -0.479 to 25.929 | 0.059 |
