# Supplementary material for: Telemonitoring at scale for hypertension in primary care: An implementation study
Source: PLoS Med. 2020 Jun 17;17(6):e1003124. doi: 10.1371/journal.pmed.1003124 (PMC7299318; doi:10.1371/journal.pmed.1003124)
Supplement: S5 Table — (DOCX) [file pmed.1003124.s014.docx]

**S5 Table. Summary statistics for the reduction between the second and last reading within 12 months in systolic BP stratified by age, gender, Scottish Index of Multiple Deprivation (SIMD) [1], and starting BP.**

|  | **N** | **Mean** | **Std Dev** | **Median** | **Lower Quartile** | **Upper Quartile** | **Min** | **Max** |
| --- | --- | --- | --- | --- | --- | --- | --- | --- |
| **<65** | 211 | 6.43 | 14.63 | 6 | -3 | 16 | -28 | 55 |
| **65+** | 188 | 6.69 | 15.80 | 6.5 | -3.5 | 13.5 | -37 | 63 |
| **Male** | 217 | 6.95 | 15.19 | 7 | -3 | 15 | -37 | 63 |
| **Female** | 182 | 6.08 | 15.19 | 5 | -3 | 15 | -34 | 53 |
| ***SIMD<5** | 70 | 7.80 | 13.41 | 6.5 | 0 | 16 | -25 | 50 |
| **SIMD 5 or more** | 329 | 6.29 | 15.53 | 6 | -3 | 15 | -37 | 63 |
| **SBP<135** | 209 | -1.18 | 11.80 | 0 | -7 | 7 | -37 | 28 |
| **SBP 135 or above** | 190 | 15.06 | 13.88 | 13 | 6 | 23 | -17 | 63 |

Higher SIMD is less deprived

[1] Scottish Government. The Scottish Index of Multiple Deprivation. 2016.
