# Supplementary material for: Telemonitoring at scale for hypertension in primary care: An implementation study
Source: PLoS Med. 2020 Jun 17;17(6):e1003124. doi: 10.1371/journal.pmed.1003124 (PMC7299318; doi:10.1371/journal.pmed.1003124)
Supplement: S6 Table — (DOCX) [file pmed.1003124.s015.docx]

**S6 Table: Clinician actions following a patient recording systolic BP< 90mmHg or >160mmHg**

|  | **Action** | **Number** |
| --- | --- | --- |
| **Systolic BP >160mmHg** | Appointment/Message sent | 4 |
|  | Increase medication | 24 |
|  | Lifestyle advice | 3 |
|  | Referral | 5 |
|  | Monitor closely | 5 |
|  | Patient refused medication increase | 2 |
|  | No action | 1 |
|  | **Total** | **44** |
| **Systolic BP <90mmHg** | Appointment/telephone consultation | 2 |
|  | No action | 1 |
|  | **Total** | **3** |
