# Supplementary material for: Telemonitoring at scale for hypertension in primary care: An implementation study
Source: PLoS Med. 2020 Jun 17;17(6):e1003124. doi: 10.1371/journal.pmed.1003124 (PMC7299318; doi:10.1371/journal.pmed.1003124)
Supplement: S7 Table — (DOCX) [file pmed.1003124.s016.docx]

**S7 Table. The change in Defined Daily Doses of BP lowering drugs in the telemonitored group in the periods before and after commencing telemonitoring.**

| **Period** | **Days** | **Aggregate DDDs (of 622 patients)** | **DDDs /patient/ day** |
| --- | --- | --- | --- |
| -182 to -135* | 47 | 56771.3 | 1.94 |
| -135 to -90 | 45 | 54476.43 | 1.95 |
| -90 to -45 | 45 | 54879.9 | 1.960697 |
| -45 to 0 | 45 | 58343.22 | 2.084431 |
| 0 to 45 | 45 | 62856.34 | 2.245671 |
| 45 to 90 | 45 | 62269.16 | 2.224693 |
| 90 to 135 | 45 | 61495.76 | 2.197062 |
| 135 to 182 | 48 | 69627.46 | 2.33211 |

* negative indicates days prior to starting telemonitoring
