# Supplementary material for: Telemonitoring at scale for hypertension in primary care: An implementation study
Source: PLoS Med. 2020 Jun 17;17(6):e1003124. doi: 10.1371/journal.pmed.1003124 (PMC7299318; doi:10.1371/journal.pmed.1003124)
Supplement: S8 Table — (DOCX) [file pmed.1003124.s017.docx]

**S8 Table: Number of prescriptions per patient in year before compared to year after, overall and stratified by age, gender, SIMD and initial BP in intervention and comparator groups**

| **INTERVENTION GROUP** | | **N** | **Mean** | **Std Dev** | **Median** | **Lower Quartile** | **Upper Quartile** | **Min** | **Max** |
| --- | --- | --- | --- | --- | --- | --- | --- | --- | --- |
| Overall | Year before | 619 | 9.63 | 6.75 | 8 | 6 | 13 | 0 | 45 |
|  | Year after | 619 | 10.69 | 6.46 | 9 | 6 | 14 | 0 | 45 |
|  | Increase | 619 | 1.06 | 4.77 | 0 | -2 | 3 | -16 | 29 |
| Age<65 | Year before | 335 | 8.44 | 6.87 | 7 | 4 | 12 | 0 | 45 |
|  | Year after | 335 | 10.22 | 6.47 | 8 | 6 | 14 | 0 | 39 |
|  | Increase | 335 | 1.77 | 5.21 | 1 | -1 | 4 | -13 | 29 |
| Age 65+ | Year before | 284 | 11.03 | 6.34 | 10 | 6 | 14 | 0 | 42 |
|  | Year after | 284 | 11.25 | 6.42 | 10.5 | 6 | 14 | 0 | 45 |
|  | Increase | 284 | 0.22 | 4.05 | 0 | -2 | 2 | -16 | 15 |
| Male | Year before | 319 | 10.17 | 7.26 | 8 | 6 | 14 | 0 | 45 |
|  | Year after | 319 | 11.46 | 7.14 | 10 | 6 | 14 | 0 | 45 |
|  | Increase | 319 | 1.29 | 5.36 | 1 | -2 | 3 | -16 | 29 |
| Female | Year before | 300 | 9.06 | 6.13 | 7 | 6 | 12 | 0 | 42 |
|  | Year after | 300 | 9.87 | 5.56 | 8 | 6 | 13 | 0 | 39 |
|  | Increase | 300 | 0.82 | 4.05 | 0 | -2 | 2 | -11 | 17 |
| SIMD<5 | Year before | 102 | 9.65 | 7.97 | 7.5 | 4 | 14 | 0 | 42 |
|  | Year after | 102 | 12.03 | 7.52 | 10 | 7 | 16 | 0 | 37 |
|  | Increase | 102 | 2.38 | 6.66 | 2 | -1 | 5 | -11 | 29 |
| SIMD 5+ | Year before | 512 | 9.64 | 6.51 | 8 | 6 | 13 | 0 | 45 |
|  | Year after | 512 | 10.40 | 6.21 | 9 | 6 | 14 | 0 | 45 |
|  | Increase | 512 | 0.77 | 4.24 | 0 | -2 | 3 | -16 | 17 |
| SBP<135 | Year before | 334 | 9.94 | 6.46 | 8 | 6 | 13 | 0 | 42 |
|  | Year after | 334 | 10.43 | 6.23 | 9 | 6 | 14 | 0 | 39 |
|  | Increase | 334 | 0.49 | 4.06 | 0 | -2 | 2 | -16 | 29 |
| SBP 135 or above | Year before | 282 | 9.23 | 7.09 | 7 | 6 | 12 | 0 | 45 |
|  | Year after | 282 | 10.97 | 6.75 | 9 | 6 | 14 | 0 | 45 |
|  | Increase | 282 | 1.74 | 5.41 | 1 | -1 | 5 | -13 | 21 |
| SBP 140 or above | Year before | 199 | 8.85 | 6.90 | 7 | 5 | 12 | 0 | 37 |
|  | Year after | 199 | 11.11 | 6.85 | 9 | 6 | 14 | 0 | 45 |
|  | Increase | 199 | 2.26 | 5.73 | 1 | -1 | 6 | -13 | 21 |
| SBP 145 or above | Year before | 131 | 9.70 | 7.64 | 8 | 5 | 14 | 0 | 37 |
|  | Year after | 131 | 12.14 | 7.56 | 10 | 7 | 17 | 0 | 45 |
|  | Increase | 131 | 2.44 | 6.21 | 2 | -2 | 6 | -13 | 21 |
| SBP 150 or above | Year before | 92 | 10.14 | 8.10 | 8 | 6 | 14 | 0 | 37 |
|  | Year after | 92 | 12.74 | 7.70 | 12 | 7 | 18 | 0 | 45 |
|  | Increase | 92 | 2.60 | 6.65 | 2 | -1.5 | 7 | -13 | 21 |

| **COMPARATOR GROUP** | | **N** | **Mean** | **Std Dev** | **Median** | **Lower Quartile** | **Upper Quartile** | **Min** | **Max** |
| --- | --- | --- | --- | --- | --- | --- | --- | --- | --- |
| Overall | Year before | 6292 | 11.18 | 8.17 | 10 | 6 | 15 | 0 | 69 |
|  | Year after | 6292 | 12.21 | 7.97 | 12 | 6 | 15 | 0 | 68 |
|  | Increase | 6292 | 1.02 | 6.36 | 0 | -2 | 3 | -54 | 56 |
| Age<65 | Year before | 2130 | 9.02 | 7.30 | 7 | 4 | 13 | 0 | 69 |
|  | Year after | 2130 | 10.91 | 7.05 | 9 | 6 | 14 | 0 | 65 |
|  | Increase | 2130 | 1.88 | 5.57 | 1 | -1 | 4 | -21 | 56 |
| Age 65+ | Year before | 4021 | 12.32 | 8.36 | 12 | 6 | 17 | 0 | 66 |
|  | Year after | 4021 | 12.92 | 8.28 | 12 | 7 | 17 | 0 | 68 |
|  | Increase | 4021 | 0.60 | 6.64 | 0 | -2 | 3 | -54 | 53 |
| Male | Year before | 2927 | 11.36 | 8.30 | 11 | 6 | 15 | 0 | 66 |
|  | Year after | 2927 | 12.36 | 7.87 | 12 | 6 | 16 | 0 | 68 |
|  | Increase | 2927 | 1.00 | 6.40 | 0 | -2 | 3 | -54 | 56 |
| Female | Year before | 3365 | 11.03 | 8.05 | 10 | 6 | 14 | 0 | 69 |
|  | Year after | 3365 | 12.07 | 8.05 | 11 | 6 | 15 | 0 | 65 |
|  | Increase | 3365 | 1.04 | 6.33 | 0 | -2 | 3 | -38 | 50 |
| SIMD<5 | Year before | 1376 | 11.58 | 8.83 | 10.5 | 6 | 15 | 0 | 69 |
|  | Year after | 1376 | 12.98 | 8.70 | 12 | 7 | 17 | 0 | 65 |
|  | Increase | 1376 | 1.40 | 6.73 | 1 | -1 | 4 | -32 | 53 |
| SIMD 5+ | Year before | 4841 | 11.11 | 7.97 | 10 | 6 | 14 | 0 | 66 |
|  | Year after | 4841 | 11.99 | 7.73 | 12 | 6 | 15 | 0 | 68 |
|  | Increase | 4841 | 0.88 | 6.22 | 0 | -2 | 3 | -54 | 56 |
| SBP<135 | Year before | 2447 | 11.80 | 8.08 | 12 | 6 | 15 | 0 | 69 |
|  | Year after | 2447 | 12.09 | 8.00 | 12 | 6 | 15 | 0 | 65 |
|  | Increase | 2447 | 0.28 | 6.40 | 0 | -2 | 2 | -54 | 56 |
| SBP 135 or above | Year before | 3845 | 10.78 | 8.20 | 9 | 6 | 14 | 0 | 66 |
|  | Year after | 3845 | 12.28 | 7.94 | 12 | 7 | 16 | 0 | 68 |
|  | Increase | 3845 | 1.50 | 6.29 | 1 | -1 | 4 | -42 | 50 |
| SBP 140 or above | Year before | 3078 | 10.61 | 8.23 | 9 | 6 | 14 | 0 | 66 |
|  | Year after | 3078 | 12.35 | 8.00 | 12 | 7 | 16 | 0 | 68 |
|  | Increase | 3078 | 1.74 | 6.46 | 1 | -1 | 4 | -42 | 50 |
| SBP 145 or above | Year before | 2141 | 10.15 | 8.43 | 8 | 5 | 14 | 0 | 66 |
|  | Year after | 2141 | 12.32 | 8.04 | 12 | 7 | 16 | 0 | 68 |
|  | Increase | 2141 | 2.17 | 6.74 | 1 | -1 | 5 | -42 | 48 |
| SBP 150 or above | Year before | 1622 | 9.96 | 8.69 | 8 | 4 | 14 | 0 | 66 |
|  | Year after | 1622 | 12.51 | 8.31 | 12 | 7 | 16 | 0 | 68 |
|  | Increase | 1622 | 2.54 | 7.00 | 2 | -1 | 6 | -42 | 48 |
