# Supplementary material for: Telemonitoring at scale for hypertension in primary care: An implementation study
Source: PLoS Med. 2020 Jun 17;17(6):e1003124. doi: 10.1371/journal.pmed.1003124 (PMC7299318; doi:10.1371/journal.pmed.1003124)
Supplement: S9 Table — (DOCX) [file pmed.1003124.s018.docx]

**S9 Table: Relevant quotations from interviews categorised according to themes**

| Themes | Quotes |
| --- | --- |
| **Health Care Professionals** | |
| Attractions | *‘Well, I already do home BP monitoring with my patients, but the system doesn’t work quite so well, so I thought this looked like a more efficient system’ (GP)*  *‘Well, I don’t go looking at them*,[Docman reports] *they come to me automatically, so they come in my workflow, my daily workflow, and that’s I think the best part of this that I don’t have to go anywhere looking for them, they just automatically come and I can assess them and compare with the previous ones and do whatever needs to be done.’(GP)*  ‘*I think the Florence system, added to what we’ve got already means that I think patients will be treated more appropriately.’ (PN)*  *‘and people like it. The only thing that I’ve heard is people saying that they like it, and in fact if there was a complaint it was somebody who was offered it and then clinically it wasn’t quite appropriate at that time because it wasn’t a priority, the patient felt hard done by because he didn’t have his monitor, so the opposite is true, people want it’* (GP)  *‘It increases our patients' awareness of hypertension and what causes it. It generally gets the patient more actively involved and encourages them to ask questions, which is great’ (PN)* |
| Practice organisation | *‘I think it seems to be well thought out. The protocols are very clear, the patients seem to…at least in the initial stages, seem to appreciate the intervention and I think going forward that I think there is a clear vision of not having patients coming in for routine hypertension, and that must be the way forward.’ (GP)*  *‘Usually weekly, because of the way I’m recruiting them in that their BPs are not perfect, so I start off weekly and then see how they get on, and then change them to monthly after that’ (PN)*  *‘that’s probably why we’ve recruited so many, we’re just making…it’s become mainstream work now. We’re happy to take full advantage of the opportunity, really.’(GP)*  *‘I just feel that this has got a lot of value for the future. We’ll see if it ends up being the standard model for managing hypertension. I’ve got a feeling that for a lot of patients it will be for the future.’(GP)* |
| Barriers to implementation | *‘Well, it took me months, actually. I just wanted to pilot patients to begin with before I knew if there was any large scale roll out….. I’d like to start delegating it to the practice nurses and healthcare assistants.’ (GP)*  *‘I was in a really busy period and it was…it is a lot of information you get, and even though somebody came out to show me, it was still a bit of a hurdle to actually get going.’(GP)*  *‘Using the Scale-Up-BP system I think at the moment is still very young, …... So recruiting patients and getting them on board has proved to be relatively straightforward. The bit I’m still not completely clear about is what I do with all that information and how I get it back, and how I manage it, because there’s not been a huge amount of it yet to be able to get a process up and running that we think is efficient.’ (PN)*  *‘They’re* [the result] *getting shared out amongst the GPs. We got to a point a few months back where it was getting a bit onerous at times.’ (HCA)* |
| Trialling at the practice level | *‘I don’t have time to do scale up with everybody. I’d have to be convinced of the value of giving somebody a monitor that needs their BP checked once a year. I suppose what I’ve done is I’ve tried to do a cross section of patients, because selfishly, I kind of feel something’s got to be in it for me’ (PN)*  *‘Dr XX took on board some of it as well, and Dr XX; but primarily it would be myself that would be coming across these patients, you know. Either for annual reviews; diabetic reviews; hypertension reviews, what have you, anyway, it would be highlighted that, for one way or another, BP wasn't being controlled’ (PN)* |
| **Patients** | |
| Self-awareness | *‘I think it’s good because it makes you realise because, you know, BPs a sort of hidden thing normally. And you are not really aware of it unless you’re uptight and fed up about something or something’s gone wrong and you are more anxious or something. You might think that’s not really going to do me much good. But I think it’s quite a good thing actually to be made more aware of.’*  *‘I think it’s sensible all round because it saves the GP or the nurse time.’* |
| Usability | *‘It was more convenient and also I think more realistic than people going into a doctor’s surgery and possibly, you know, hurrying because they are later, you know, and being all uptight and maybe having an increased reading.’* |
