# Supplementary material for: Telemonitoring at scale for hypertension in primary care: An implementation study
Source: PLoS Med. 2020 Jun 17;17(6):e1003124. doi: 10.1371/journal.pmed.1003124 (PMC7299318; doi:10.1371/journal.pmed.1003124)
Supplement: S10 Table — (DOCX) [file pmed.1003124.s019.docx]

**S10 Table: Barriers and facilitators identified during the evaluation phase and potential solutions suggested by participants**

|  | **Challenges identified in Scale-Up-BP process** | **Potential implementation strategies** |
| --- | --- | --- |
| **Patients** | - Inconvenience of face-to-face reviews | Consider letting patients self-register for the system. Promote the advantages direct to patients |
|  | - Lack of confidence for less IT literate | Helpline; funded in-practice support |
| **Professionals** | - Perception that IT is complex | Improved training. Help line |
|  | - Perception that telehealth increases workload | Publish evidence from this study suggesting face-to-face appointment time may be saved |
| **Practice organisation** | - Docman solution allows BP results to be managed with normal document handling, but does not put summary BP into electronic record in an auditable fashion | Further development of reporting software to allow this |
|  | - Patients not always contactable by phone and response to raised BP is to ask reception to write a letter to patient to call the patient in | Explore asynchronous methods of connecting with patients. Use of apps. Encourage arrangement of telephone reviews |
|  | - Additional time needed to add patients to system | Backfill payment to practices to pay for additional HCP time  Support staff sent to practices to help. Simplification of registration |
| **Policy and wider context** | - Potential lack of top-down encouragement Lack of clinical leadership on the ground | Identify local champions to encourage implementation  Promotion from government/health boards  Target setting for telemonitoring adoption |
|  | - Potential lack of equity | Provision of sphygmomanometers at no cost. Setting inclusion targets. |
