# Supplementary material for: Telemonitoring at scale for hypertension in primary care: An implementation study
Source: PLoS Med. 2020 Jun 17;17(6):e1003124. doi: 10.1371/journal.pmed.1003124 (PMC7299318; doi:10.1371/journal.pmed.1003124)
Supplement: S11 Table — (DOCX) [file pmed.1003124.s020.docx]

**S11 Table: Change in BP in intervention groups of comparable UK telemonitoring trials to Scale-Up BP**

| **Name and number in telemonitoring group** | **year** | **Average age** | **Intervention length (months)** | **Measurement** | **Minimum entry SBP** | **Baseline SBP telemonitoring intervention group** | **Final SBP** | **Difference** | **Baseline DBP** | **Final DBP** | **Difference**  **mmHg ( at number of months)** |
| --- | --- | --- | --- | --- | --- | --- | --- | --- | --- | --- | --- |
| TASMINH2 [1]  N=250 | 2010 | 66.6 | 12 | Mean of second two of 3 office BPs | SBP>140 or DBP >90 | 152.1 | 134.9 | 17.2 | 85 | 79.6 (6m)  77.4 (12m) | 5.4 (6m)  7.6 (12m) |
| HITS [2]  n=200 | 2013 | 60.5 | 6 | Mean day-time ambulatory BP | SBP>135 or DBP >85 | 146.2 | 140 | 6.2 | 87.1 | 83.4 | 3.7 (6m) |
| Telescot Diabetes [3]  n=146 | 2014 | 60.5 | 9 | Mean day-time ambulatory BP | none | 133.7 | 131.0 | 2.7 | 78.5 | 76.2 | 2.3 (9m) |
| Telescot Stroke TIA [4]  n=40 | 2015 | 69.9 | 6 | Mean day-time ambulatory BP | SBP>130 | 144.1 | 133.9 | 10.2 | 79.2 | 75.2 | 4.0 (6m) |
| TASMINH4 [5]  n=393 | 2018 | 67 | 12 | Mean of second two of 3 office BPs | SBP>140 or DBP >90 | 153.3 | 139 (6 months)  136 (12months) | 14.3 (6 months)  17.3 (12 months) | 85.5 | 84.3 ( 6m)  84.2 (12m) | 1.2 (6m)  1.3 (12m) |
| **Scale-Up BP**  n=399 | 2019 | 62.5 | 6-12 | Home monitored BP | None (diagnosed HBP) | 133.9 | 127.4 | 6.6 | 80.9 | 76.6 | 4.2 (6-12m) |
| **Scale-Up BP participants with baseline >135mmHg**  n=190 | 2019 | 63.4 | 6-12 | Home monitored BP | Subset of those with starting BP>135 | 147.7 | 132.2 | 15.5 | 85.5 | 78.1 | 7.4 (6-12m) |

1. McManus RJ, Mant J, Bray EP, Holder R, Jones MI, Greenfield S, et al. Telemonitoring and self-management in the control of hypertension (TASMINH2): a randomised controlled trial. Lancet. 2010;376(9736):163-72. doi: 10.1016/S0140-6736(10)60964-6. PubMed PMID: 20619448.

2. McKinstry B, Hanley J, Wild S, Pagliari C, Paterson M, Lewis S, et al. Telemonitoring based service redesign for the management of uncontrolled hypertension: multicentre randomised controlled trial. Bmj. 2013;346:f3030. doi: 10.1136/bmj.f3030. PubMed PMID: 23709583; PubMed Central PMCID: PMC3663293.

3. Wild SH, Hanley J, Lewis SC, McKnight JA, McCloughan LB, Padfield PL, et al. Supported Telemonitoring and Glycemic Control in People with Type 2 Diabetes: The Telescot Diabetes Pragmatic Multicenter Randomized Controlled Trial. PLoS Med. 2016;13(7):e1002098. doi: 10.1371/journal.pmed.1002098. PubMed PMID: 27458809; PubMed Central PMCID: PMCPMC4961438.

4. Hanley J, Fairbrother P, Krishan A, McCloughan L, Padfield P, Paterson M, et al. Mixed methods feasibility study for a trial of blood pressure telemonitoring for people who have had stroke/transient ischaemic attack (TIA). Trials. 2015;16:117. doi: 10.1186/s13063-015-0628-y. PubMed PMID: 25873155; PubMed Central PMCID: PMCPMC4404620.

5. McManus RJ, Mant J, Franssen M, Nickless A, Schwartz C, Hodgkinson J, et al. Efficacy of self-monitored blood pressure, with or without telemonitoring, for titration of antihypertensive medication (TASMINH4): an unmasked randomised controlled trial. Lancet. 2018;391(10124):949-59. doi: 10.1016/S0140-6736(18)30309-X. PubMed PMID: 29499873; PubMed Central PMCID: PMCPMC5854463.
