# Supplementary material for: Telemonitoring at scale for hypertension in primary care: An implementation study
Source: PLoS Med. 2020 Jun 17;17(6):e1003124. doi: 10.1371/journal.pmed.1003124 (PMC7299318; doi:10.1371/journal.pmed.1003124)
Supplement: S1 Text — (DOCX) [file pmed.1003124.s021.docx]

**S1 Text : The Scale-Up BP Intervention**

The intervention is open to people whose blood pressure (BP) is being managed by the practice who have (or whose carer has) a mobile phone and are able to text. Patients are asked to bring their mobile phone with them to a dedicated appointment. Patients are given a British Hypertension Society approved sphygmomanometer and shown how to use it, registered with a third party telemonitoring system (in this case Florence Telehealth) and the NHS Lothian reporting system (a novel program which extracts data from the Florence system for export to the NHS electronic medical record) by the health care practitioner and an action plan is agreed.

Patients are asked to check their BP with a frequency determined by their health care practitioner based on their current level of control. Texts are sent at the times of day and days of the week that suit them best reminding them to check their BP, key-in the results and text them back to a third party website. If well controlled, then monthly or bi-monthly BP values were requested, however some patients and clinicians prefer to send a series of BP measures over a week every six months. Patients are sent an automated text message to tell them the BP reading has been received and if it is within target and, if high, if they should repeat it. (See Fig S1) Targets are based on National Institute for Clinical Excellence guidelines [1]. Repeatedly elevated readings trigger advice to follow their action plan (e.g. contact the surgery routinely by telephone if moderately high or more urgently for very high readings (systolic >180). Patients are expected to take an active role in their care and it is made clear that action on very high readings is their responsibility as the clinician will not necessarily see the report right away.

**S1 Fig: The Scale-Up System**

Software to interrogate the third-party web -based dataset, developed in association with NHS Lothian, summarise the data into monthly, three monthly or six monthly reports (see S2 Fig ) and are sent to the GP through their standard document handling service (Docman). The frequency of the report is chosen by the health care practitioner depending on the current level of control. The report is structured to provide graphical and tabular information on the progress of the patients’ blood pressure along with an average of the last five blood pressures, a clear statement as to whether or not the agreed target has been met and an appropriate exhortation to consider intervention if required. The report is viewed by the GP or practice nurse alongside other communications such as laboratory results or hospital letters. Any alterations in medication can be intimated to the patient by telephone, email, letter or text according to preference (of practice and patient), with patients only invited to attend the surgery if blood tests or physical examination was required.

1. National Institute for Clinical Excellence. Hypertension in adults: diagnosis and management. Clinical guideline [CG127]. 2011.
