# Supplementary material for: Telemonitoring at scale for hypertension in primary care: An implementation study
Source: PLoS Med. 2020 Jun 17;17(6):e1003124. doi: 10.1371/journal.pmed.1003124 (PMC7299318; doi:10.1371/journal.pmed.1003124)
Supplement: S2 Text — (DOCX) [file pmed.1003124.s022.docx]

**S2 Text : Qualitative exploration of patient and professional perceptions methods**

In the eight practices, we undertook semi-structured face-to-face interviews with patients, GPs, practice nurses and HCAs, to explore perceptions of the telemonitoring intervention in terms of ease of use, organisational adoption and adaptation, barriers, facilitators and potential improvements to the implementation of Scale-Up BP. These interviews took place in the first six months of the new service being launched. The topic guide, based on our previous work [2] and theoretical considerations [3, 4], was reviewed and iteratively refined. Patients were interviewed at home and HCPs at their workplace. The interviews were audio-recorded, transcribed and imported into NVivo version 11 (QSR International, Doncaster, Victoria, Australia). Interviews continued until data saturation was believed to have been achieved. After duplicate coding of a sample of the interview transcripts, an agreed coding framework was used for thematic analysis, using an interpretive descriptive approach [5]. Constant comparison was undertaken to ensure that the analysis represented all views.

We observed and recorded as field notes the implementation process including how patients were recruited, how Docman reporting was managed and the action taken if patients submitted a critical BP reading. Specifically we noted what action was recorded, if any, when patient values gave an average systolic BP >160mmHg or <90mmHg for up to 25 patients/practice.

1. Hanley J, Ure J, Pagliari C, Sheikh A, McKinstry B. Experiences of patients and professionals participating in the HITS home blood pressure telemonitoring trial: a qualitative study. BMJ open. 2013;3(5). doi: 10.1136/bmjopen-2013-002671. PubMed PMID: 23793649; PubMed Central PMCID: PMC3657666.

2. Greenhalgh T, Robert G, Macfarlane F, Bate P, Kyriakidou O. Diffusion of innovations in service organizations: systematic review and recommendations. The Milbank quarterly. 2004;82(4):581-629. Epub 2004/12/15. doi: 10.1111/j.0887-378X.2004.00325.x. PubMed PMID: 15595944; PubMed Central PMCID: PMCPmc2690184.

3. Michie S, van Stralen MM, West R. The behaviour change wheel: A new method for characterising and designing behaviour change interventions. Implement Sci. 2011;6(1):42. doi: 10.1186/1748-5908-6-42.

4. Thorne S. Interpretive description. Walnut Creek, CA: Left Coast Press; 2008.

5. Miech EJ, Rattray NA, Flanagan ME, Damschroder L, Schmid AA, Damush TM. Inside help: An integrative review of champions in healthcare-related implementation. SAGE Open Med. 2018;6:2050312118773261. doi: 10.1177/2050312118773261. PubMed PMID: 29796266; PubMed Central PMCID: PMCPMC5960847.
