# Supplementary material for: Telemonitoring at scale for hypertension in primary care: An implementation study
Source: PLoS Med. 2020 Jun 17;17(6):e1003124. doi: 10.1371/journal.pmed.1003124 (PMC7299318; doi:10.1371/journal.pmed.1003124)
Supplement: S3 Text — (DOCX) [file pmed.1003124.s023.docx]

**S3 Text: Perceptions of the implementation of Scale-UP BP**

*Observation*

Most practices initially extended their appointment times in order to consent, train and register patients although with experience this was reduced. Some practices allocated time for triaging telemonitoring data to an individual (e.g. nurse/HCA) passing abnormal results to the duty doctor, while in other practices incoming reports were directed straight to the responsible GP . The time taken to review a Docman report varied from several seconds where no action was needed, to up to 5 minutes if a clinical change was made and patient informed.

The preferred approach for contacting patients using Scale-Up BP was by telephone with occasional texting. GPs were not happy with email communication as there was no natural closure to the consultation. Some patients expected a face-to-face discussion if their BP was raised to a critical value and made appointments although others were happy to speak over the telephone.

*Clinician and patient views of the implementation*

We interviewed seven GPs, nine practice nurses, one health care assistant and 21 patients. Themes and supporting quotes are presented in Table S9. HCPs liked the summary BP report (via Docman), which was integrated within the existing result managing systems with clear action prompts.

Practice staff valued the clear protocols provided with Scale-Up BP, the training to use the system, and felt supported by facilitators. Technical problems were quickly resolved; however some perceived the initial registration and data entry to be complex which led to some negative comments during practice recruitment. Time for setting up systems for identifying and inviting patients and re-organising work schedules to accommodate this was challenging. For some finding appropriate staff to lead the new service, and a lack of IT skills were problems. Some HCPs required additional training. S10 Table lists the challenges and potential solutions to deploying and sustaining BP telemonitoring at scale raised during the study.

In the early adopter practices it was clear that implementation was an evolving process, systems were developed for patient identification and recruitment, but were adapted as patient numbers grew in terms of how frequently BP readings were requested and the frequency of Docman reports. For example, some practice evolved systems whereby an HCA screened reports sending only those outside pre-determined parameters to clinicians whilst others engaged the whole clinical team in reviewing results.

GP champions for telehealth played an important role not only in their practice, but more widely in NHS Lothian. Non-participating practices became aware of Scale-Up BP and the fact that it was being used successfully through the use of newsletters and local presentations.

With respect to patients, HCPs perceived that telemonitoring supported self-management and improved care through more accurate readings and appropriate treatment adjustments. They also reported a sense that patients liked being part of Scale-Up BP, and embraced the technology. Patients said it saved them time, were reassured that their results were being monitored regularly, and felt an increased sense of empowerment and awareness of their condition.
