# Supplementary material for: Telemonitoring at scale for hypertension in primary care: An implementation study
Source: PLoS Med. 2020 Jun 17;17(6):e1003124. doi: 10.1371/journal.pmed.1003124 (PMC7299318; doi:10.1371/journal.pmed.1003124)
Supplement: Attachment — Submitted filename: hammersley.pdf [file pmed.1003124.s024.pdf]

This paper presents a mixed methods study of implementing monitoring hypertension via telemedicine.

I was asked for a statistical report and I interpret that to include all aspects of the design and conduct of the study.

Points of detail

Page 7 I wonder whether for an international audience some more detail about Lothian might be helpful so they can evaluate how generalisable this might be to their setting?

Page 9 Some of these covariates are inherently continuous but have been categorised. Categorising an essentially continuous variable wastes information (Altman and Royston, 2006; Royston et al., 2006) and leads to models which are often implausible as they predict the effect remaining flat within categories and then jumping to a new value at the category boundary. In fact later (page 10) they do seem to have been modelled with splines so this seems inconsistent.

Page 10 Why would it be a good idea to exclude long times? If you want to look at resource use they are still relevant surely? I am not that familiar with the distribution of consultation times but times in general are positively skewed so it would be a good idea to look at regression diagnostics and report the results.

Page 10 The authors state here that they have not presented formal statistical tests but in fact in places these are presented, page 14 being an example, so this needs some explanation.

Page 15 A possible explanation for those with higher initial readings falling more is regression to the mean of course. Figure 2 is helpful here as it suggests a continued fall after month 1.

Points of more substance

Overall structure

The authors have conducted two studies which are inter–linked. I feel the description of these would benefit from a clearer separation for the reader of what comes from which part. I might divide each section (methods, results)

clearly into two and signpost them that way but I am sure there are other ways to do it.

Study sizes

The study size is variously described as 3200 (page 2), nearly 4000 (page 10), 9966 (page 24). Similarly for the nested sub–study we have 905, 430, 655, 118 and 399. I can see how this happens for the sub–study but in both cases it raises the question of whether some bias is introduced by the changes in sample size and how it affects generalisability. I am not too convinced by the authors' argument that their goal is solely description since they clearly hope that their successes in Lothian will generalise to other settings otherwise they would not be submitting it to an international journal.

Summary

Getting a complex intervention to make the jump from the RCT laboratory to everyday life is hard and this study is an impressive achievement but needs a bit more explanation.

Michael Dewey

References

D G Altman and P Royston. The cost of dichotomising continuous variables. *British Medical Journal*, 332:1080, 2006.

P Royston, D G Altman, and W Sauerbrei. Dichotomizing continuous predictors in multiple regression: a bad idea. *Statistics in Medicine*, 25:127–141, 2006.
